# In silico analysis excavates potential biomarkers by constructing miRNA-mRNA networks between non-cirrhotic HCC and cirrhotic HCC

**DOI:** 10.1186/s12935-019-0901-3

**Published:** 2019-07-18

**Authors:** Bisha Ding, Weiyang Lou, Jingxing Liu, Ruohan Li, Jing Chen, Weimin Fan

**Affiliations:** 1 Program of Innovative Cancer Therapeutics, Division of Hepatobiliary and Pancreatic Surgery, Department of Surgery, First Affiliated Hospital, College of Medicine, Key Laboratory of Combined Multi-Organ Transplantation, Ministry of Public Health, Key Laboratory of Organ Transplantation, Zhejiang University, Hangzhou, 310003 Zhejiang Province China; 2 Department of Intensive Care Unit, Changxing People’s Hospital of Zhejiang Province, Huzhou, 313100 Zhejiang, China; 3 Department of Critical Care Medicine, The Second Affiliated Hospital of Xi’an Jiaotong University, Xi’an, 710004 Shanxi China; 4 First Affiliated Hospital of Jiaxing University, Jiaxing, 314000 Zhejiang, China

**Keywords:** Gene, miRNA, Non-cirrhotic, Cirrhotic, HCC

## Abstract

**Background:**

Mounting evidences have demonstrated that HCC patients with or without cirrhosis possess different clinical characteristics, tumor development and prognosis. However, few studies directly investigated the underlying molecular mechanisms between non-cirrhotic HCC and cirrhotic HCC.

**Methods:**

The clinical information and RNA-seq data were downloaded from The Cancer Genome Atlas (TCGA) database. Differentially expressed genes (DEGs) of HCC with or without cirrhosis were obtained by R software. Functional annotation and pathway enrichment analysis were performed by Enrichr. Protein–protein interaction (PPI) network was established through STRING and mapped to Cytoscape to identify hub genes. MicroRNAs were predicted through miRDB database. Furthermore, correlation analysis between selected genes and miRNAs were conducted via starBase database. MiRNAs expression levels between HCC with or without cirrhosis and corresponding normal liver tissues were further validated through GEO datasets. Finally, expression levels of key miRNAs and target genes were validated through qRT-PCR.

**Results:**

Between 132 non-cirrhotic HCC and 79 cirrhotic HCC in TCGA, 768 DEGs were acquired, mainly involved in neuroactive ligand-receptor interaction pathway. According to the result from gene expression analysis in TCGA, *CCL19*, *CCL25*, *CNR1*, *PF4* and *PPBP* were renamed as key genes and selected for further investigation. Survival analysis indicated that upregulated *CNR1* correlated with worse OS in cirrhotic HCC. Furthermore, ROC analysis revealed the significant diagnostic values of *PF4* and *PPBP* in cirrhotic HCC, and *CCL19*, *CCL25* in non-cirrhotic HCC. Next, 517 miRNAs were predicted to target the 5 key genes. Correlation analysis confirmed that 16 of 517 miRNAs were negatively regulated the key genes. By detecting the expression levels of these key miRNAs from GEO database, we found 4 miRNAs have high research values. Finally, potential miRNA-mRNA networks were constructed based on the results of qRT-PCR.

**Conclusion:**

In silico analysis, we first constructed the miRNA-mRNA regulatory networks in non-cirrhotic HCC and cirrhotic HCC.

**Electronic supplementary material:**

The online version of this article (10.1186/s12935-019-0901-3) contains supplementary material, which is available to authorized users.

## Background

Hepatocellular carcinoma (HCC) ranks the third leading cause of cancer death and the sixth most frequently diagnosed neoplasm among the world [1]. 782,500 new liver cancer patients were diagnosed and 745,500 deaths occurred worldwide in 2012, while China occupy half number of the total cases [2]. The occurrence of HCC is strongly correlated with etiological factors, including virus infection related-cirrhosis, alcohol abuse related-cirrhosis, non-alcoholic fatty liver disease (NAFLD) related-cirrhosis [2–6]. It’s widely known that HCC and cirrhosis are two common progressive fibrotic liver diseases causing death and the progression of HCC is closely related with cirrhosis, as the majority of HCC patients are accompanied with liver cirrhosis [7, 8]. However, there is still about 20% of HCC cases arising in non-cirrhotic livers, with limited study [9, 10]. To date, more and more analyses revealed that HCC patients with or without highly cirrhotic liver have different pathogenetic backgrounds, clinical characteristics, tumor development, prognosis and surveillance indicators, also attributed to different risk factors [4, 11–15]. Although a lot of time and efforts have been placed to well understanding HCC, the exact mechanisms about the difference and connection of non-cirrhotic HCC and cirrhotic HCC remain unclarified and require urgent consideration. Furthermore, effective treatments for HCC including cirrhotic HCC and non-cirrhotic HCC are unavailable [3].

Messenger RNA is a large family of RNA molecules that encode proteins, convey genetic information. MicroRNAs (miRNAs) are a class of small non-coding RNA molecules (containing about 22 nucleotides) that conversely regulate gene expression at the post-transcriptional level [16, 17]. Gene and miRNA play significant roles in various essential tumor biological processes by constructing miRNA-mRNA networks [18, 19].

In this study, we aim to excavate different clinical pathological of non-cirrhotic HCC and cirrhotic HCC, identify dysregulated genes between HCC with or without cirrhotic, and build miRNA-mRNA networks by using clinical data and RNA-seq data in The Cancer Genome Atlas (TCGA) database. Based on bioinformatic analysis and experimental validation, the key miRNA-mRNA interactions in non-cirrhotic HCC and cirrhotic HCC were further validated, and provided us a new insight into the mechanisms, thus developing effective therapeutic strategies for HCC.

## Materials and methods

### Selecting non-cirrhotic and cirrhotic HCC patients and screening DEGs between non-cirrhotic and cirrhotic HCC

The clinical pathological information and raw expression data were downloaded from The Cancer Genome Atlas (TCGA) database (https://cancergenome.nih.gov/). We only selected the samples which contain both clinical information and RNA-seq expression data, and subdivided these patients into two groups (non-cirrhotic HCC group and cirrhotic HCC group) in terms of the Ishak score [20]. Data were normalized and the differentially expression genes (DEGs) between two groups were both analyzed by edgeR package (http://bioconductor.org/packages/edgeR/) in R software. The |fold change (FC)| > 2 and p-value < 0.05 were set as restricted condition to identify DEGs.

### Gene ontology annotation and kyoto encyclopedia of genes and genomes pathway enrichment analysis

The Enrichr database (http://amp.pharm.mssm.edu/Enrichr/) was used to perform functional annotation and pathway enrichment analysis, including Gene Ontology annotation (GO) and kyoto encyclopedia of genes and genomes (KEGG) pathway analysis [21, 22].

### Construction and analysis of PPI network and miRNA-mRNA interaction networks

The DEGs were entered into STRING database (https://string-db.org/) to gain significant functional associations among genes [23]. The minimum required interaction score set as 0.4. Then, the string_interactions.tsv was downloaded from STRING and mapped to Cytoscape software (version 3.7.0) in order to find hub genes. In addition, expression levels analysis between HCC and corresponding normal tissues were performed to validate the selected hub genes. 5 of 15 hub genes were selected for the next research. Survival analysis was also performed to validate the 5 key genes. Besides, the target miRNAs of the 5 selected key genes were predicted via miRDB database (http://mirdb.org/miRDB/) [24, 25]. Next, we further screened the correlation between predicted miRNAs and target key genes based on starBase database (http://starbase.sysu.edu.cn/panCancer.php) [26]. Only those miRNAs that negatively correlated with target genes and the |r| value > 0.1, p value < 0.05 were chosen for the following investigation. Then, we confirmed the expression of those key miRNAs between liver normal tissues and HCC with or without cirrhosis according to GSE10694 dataset from the National Center for Biotechnology Information (NCBI) GEO database by using an online tool, namely GEO2R (https://www.ncbi.nlm.nih.gov/geo) [27].

### Clinical samples

20 cases of HCC samples including 10 non-cirrhotic HCC and 10 cirrhotic HCC clinical tissues and corresponding normal liver tissues were obtained from 20 patients who had undergo surgery from 2017 to 2018 at the First Affiliated Hospital of Zhejiang University (Hangzhou, China). The study was already obtained the informed consent from each patient and approved by the Ethics Committee of the First Affiliated Hospital of Zhejiang University.

### RNA extraction and quantitative polymerase chain reaction (qRT-PCR)

RNA extraction and qRT-PCR were performed as we previously described [22, 28–30]. Simply, total RNA was extracted from HCC clinical samples by using RNAiso plus Reagent (TaKaRa, Kusatsu, Japan). PrimeScript RT Reagent Kit (TaKaRa, RR0037A) and SYBR Premix Ex Taq (TaKaRa, RR420A) were used to perform qRT-PCR. Gene level was normalized to GAPDH and miRNA level was normalized to U6 gene expression, then relative expression level was analyzed using 2^−ΔΔCT^ method. mRNA primers (Additional file 1: Table S1) were purchased from BGI. miRNA reverse transcription primers were synthesized by RiboBio Co. Ltd (Guangzhou, China).

### Statistical analysis

Statistical analysis was conducted by GraphPad prism software (version 7.0.3), and the results were shown as mean ± SD. Differences between non-cirrhotic HCC and cirrhotic HCC group were analyzed using unpaired Student’s *t*-test. Chi square test was employed to assess the relationship between cirrhotic HCC or non-cirrhotic HCC patients’ clinical features and expression of key mRNAs and miRNAs. Only a two-tailed value of p < 0.05 was considered as statistically significant.

## Results

### Screening and analyzing differentially expressed genes between non-cirrhotic and cirrhotic HCC

**Fig. 1:**
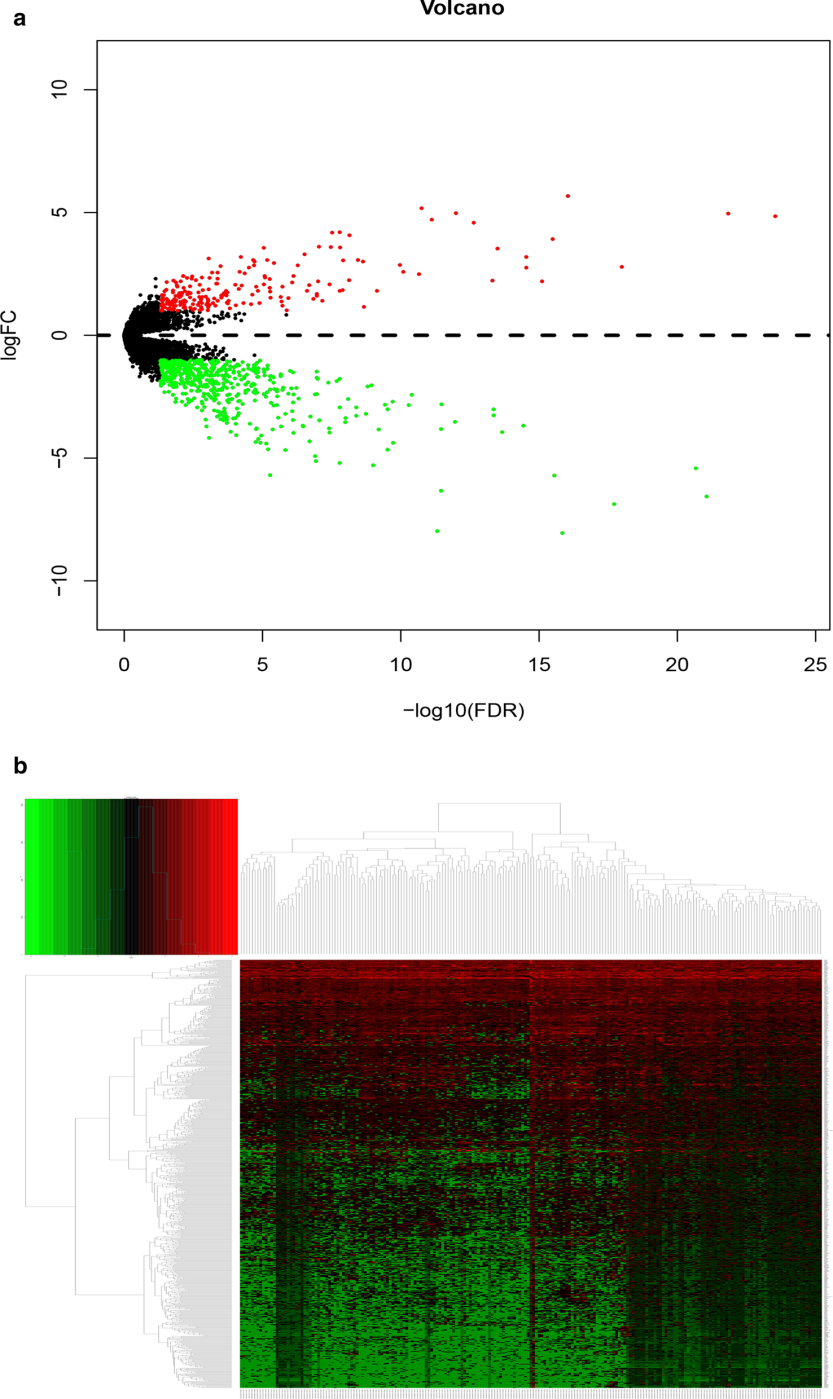
DEGs between cirrhotic HCC and cirrhotic HCC. **a** Volcano plot of DEGs between non-cirrhotic HCC and cirrhotic HCC. The red dots represent upregulated genes in cirrhotic HCC (n = 206), the green dots represent downregulated genes in cirrhotic HCC (n = 562), while the black dots represent genes that are not differentially expressed between two groups. **b** The heatmap of differential expressed genes between HCC with or without cirrhosis

A total of 377 HCC patients were found in TCGA database. Among these patients, only 218 cases contain the Ishak score in clinical information. We excluded 159 cases that lack Ishak score information and divided the rest 218 cases into two groups based on their Ishak score. The patients with scores equal or higher than 5 were included in the cirrhotic HCC group (n = 81), patients with lower scores than 5 were included in the non-cirrhotic group (n = 137). Besides, we found 6 of 377 patients lack information on gene expression, finally we choose 132 non-cirrhotic HCC and 79 cirrhotic HCC patients for further analysis, as they contain both enough clinical information and gene expression data. In the next step, based on edgeR package analysis and the cut-off criteria (fold Change ≥ 2, p value ≤ 0.05), 768 differentially expressed genes between non-cirrhotic HCC and cirrhotic HCC were found, including 206 upregulated and 562 downregulated genes (Fig. 1). In addition, clinical characteristics of non-cirrhotic HCC and cirrhotic HCC patients in TCGA were collected, including gender, age at diagnosis, TNM stage, pathologic stage, vascular invasion, HBV infection and HCV infection (Table 1). The analysis showed that the status of gender, age at diagnosis, T stage, pathologic stage, HBV infection and HCV infection were obviously different between cirrhotic HCC and non-cirrhotic HCC in TCGA (p < 0.05).

**Table 1:** Characteristics of non-cirrhotic HCC and cirrhotic HCC patients in TCGA

Variables	Non-cirrhotic HCC case (n = 132)	Cirrhotic HCC case (n = 79)	p value
Gender			
Male	82	61	0.023
Female	50	18	
Age at diagnosis			
≥ 60	80	36	0.042
< 60	52	42	
NA	0	1	
T stage			
T1/T2	97	70	0.011
T3/T4	33	8	
TX	1	0	
NA	1	1	
N stage			
N0	91	57	0.681
N1	1	1	
NX	40	20	
NA	0	1	
M stage			
M0	90	62	0.134
M1	4	0	
MX	38	17	
Pathologic stage			
I/II	91	67	0.013
III/IV	33	9	
NA	8	3	
Vascular invasion			
None	87	52	0.800
Micro	33	22	
Macro	6	2	
NA	6	3	
HBV infection			
Yes	38	36	0.014
No	88	40	
NA	6	3	
HCV infection			
Yes	14	20	0.005
No	112	56	
NA	6	3	

### GO and KEGG analysis

**Fig. 2:**
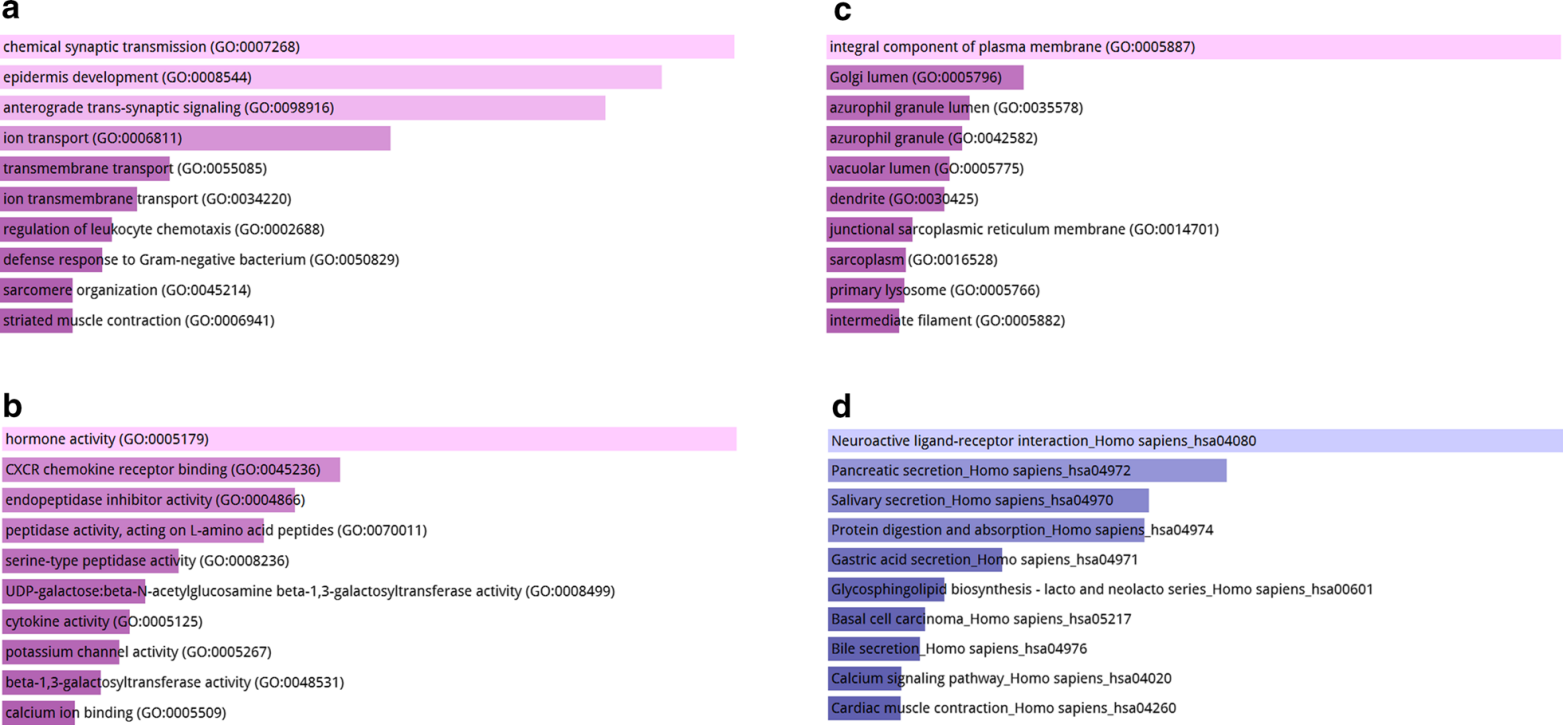
GO function analysis and KEGG pathway enrichment analysis between non-cirrhotic HCC and cirrhotic HCC. **a** Biological process (BP) analysis; **b** cellular component (CC) analysis; **c** molecular function (MF) analysis; **d** KEGG pathway enrichment analysis of differential expressed genes between non-cirrhotic HCC and cirrhotic HCC

To understand the potential biological roles of these differentially expressed genes, three categories of GO functional annotation analysis, containing biological process (BP), cellular component (CC) and molecular function (MF), were analyzed. As shown in Fig. 2a–c, DEGs are significantly enriched in chemical synaptic transmission, epidermis development, anterograde trans-synaptic signaling in the BP category, hormone activity, CXCR chemokine receptor binding, endopeptidase inhibitor activity in the CC category and integral component of plasma membrane, Golgi lumen, azurophil granule lumen in the MF category. KEGG enrichment analysis for these DEGs revealed that neuroactive ligand-receptor interaction, pancreatic secretion and salivary secretion are significantly enriched pathways (Fig. 2d).

### Identify important models and hub genes and validate expression levels, clinical characteristics, prognostic and diagnostic values of the key genes

**Fig. 3:**
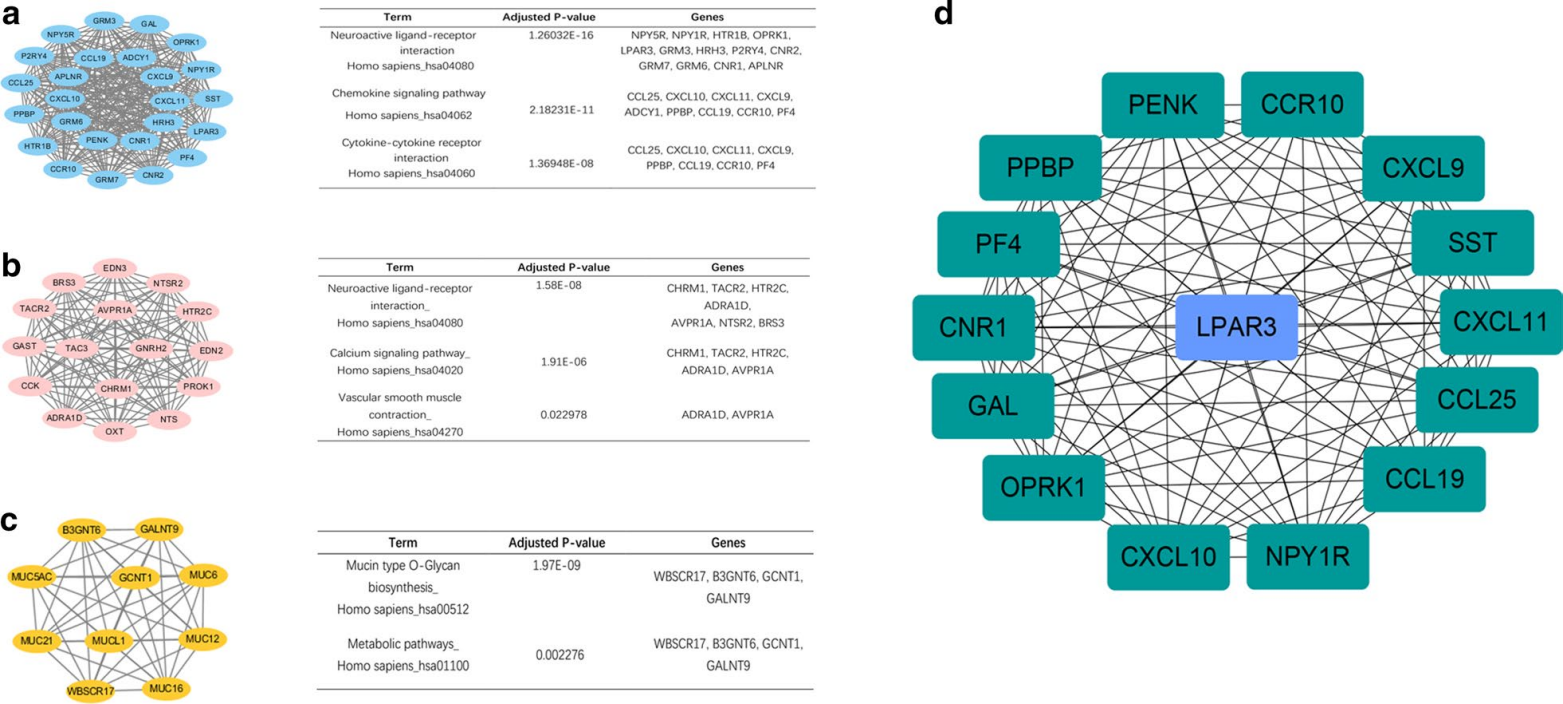
MCODE analysis, KEGG pathway enrichment analysis and PPI network of the hub genes

**Fig. 4:**
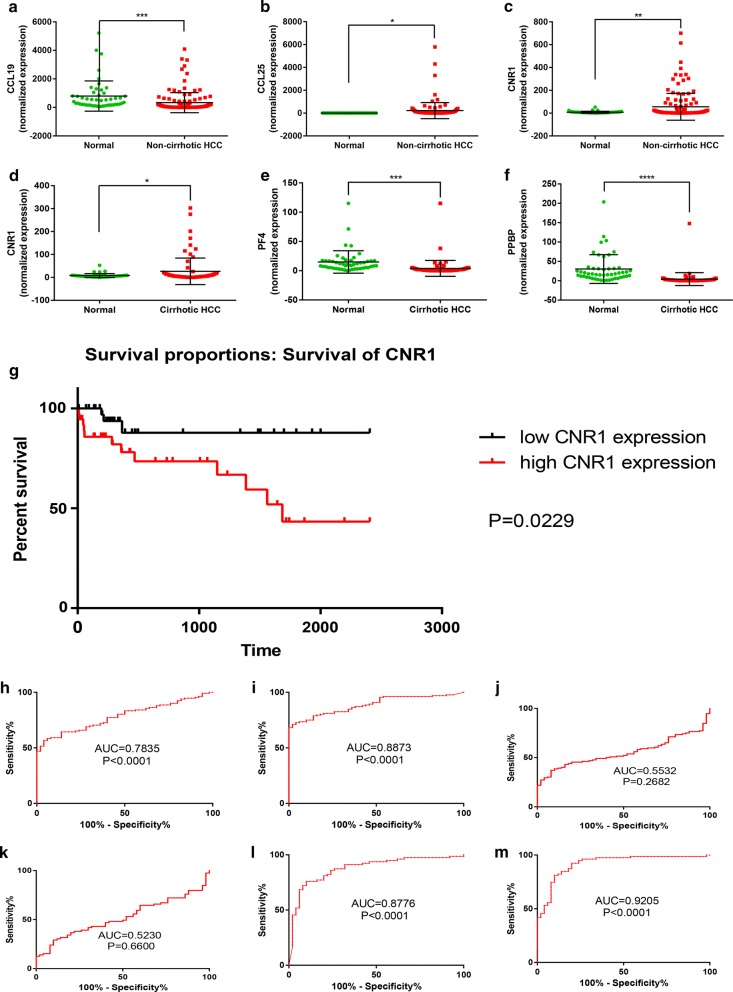
Expression analysis, survival analysis and ROC analysis of the 5 key genes between non-cirrhotic HCC and corresponding normal tissues. **a** *CCL19* expression between non-cirrhotic HCC and normal tissues in TCGA; **b** *CCL25* expression between non-cirrhotic HCC and normal tissues in TCGA; **c** *CNR1* expression between non-cirrhotic HCC and normal tissues in TCGA; **d** *CNR1* expression between cirrhotic HCC and normal tissues in TCGA; **e** *PF4* expression between cirrhotic HCC and normal tissues in TCGA; **f** *PPBP* expression between cirrhotic HCC and normal tissues in TCGA; **g** *CNR1* survival analysis in cirrhotic HCC; **h** *CCL19* ROC analysis in non-cirrhotic HCC; **i** *CCL25* ROC analysis in non-cirrhotic HCC; **g** *CNR1* ROC analysis in non-cirrhotic HCC; **k** *CNR1* ROC analysis in cirrhotic HCC; **l** *PF4* ROC analysis in cirrhotic HCC; **m** *PPBP* ROC analysis in cirrhotic HCC

The DEGs with combined scores higher than 0.4 were selected to construct PPI network using STRING database (Additional file 2: Figure S1). Subsequently, the entire PPI network was analyzed through MCODE, and top three modules were selected for further KEGG pathway enrichment analysis. As shown in Fig. 3, the top three module genes were mostly involved in neuroactive ligand-receptor interaction and mucin type O-Glycan biosynthesis. We set the screening option as Degree Cutoff = 2, Node Score Cutoff = 0.2, K-Core = 4 and Max. Depth = 100. In the next step, we screened out the top 15 hub nodes ranked by the MCC using CytoHubba plugin, as presented in Fig. 3d. The top 15 hub genes were as follows: *LPAR3*, *CXCL9*, *CXCL10*, *CXCL11*, *PF4*, *PPBP*, *CCL19*, *SST*, *GAL*, *NPY1R*, *CCL25*, *CCR10*, *PENK*, *OPRK1* and *CNR1*. Additionally, we determined the expression levels of the top 15 hub genes between HCC tissues (non-cirrhotic HCC tissues or cirrhotic HCC tissues) and HCC normal tissues in TCGA. *PF4*, *PPBP* were found to be significantly downregulated in cirrhotic HCC than that in paracancerous tissues, while *CCL25* was statistically upregulated in non-cirrhotic HCC compared with corresponding normal tissue. Besides, *CCL19* was significantly downregulated in non-cirrhotic HCC compared with corresponding normal tissues. Additionally, the expression of *CNR1* was higher in both cirrhotic HCC and non-cirrhotic HCC tissues compared with normal controls (Fig. 4).

In the next step, we validated the prognostic values of 5 key genes in cirrhotic HCC and non-cirrhotic HCC. We found that all of the non-cirrhotic HCC patients in TCGA contain with completely survival information. However, 1 of 79 cirrhotic HCC patient lack of information on survival, finally we selected 132 non-cirrhotic HCC and 78 cirrhotic HCC patients for survival analysis. Only high *CNR1* expression was found to be related to unfavorable overall survival (OS) in cirrhotic HCC (Fig. 4). In addition, a ROC curve (X-axis: 100%-Specificity%; Y-axis: Sensitivity%) was used to estimate the diagnostic values of 5 key genes in HCC with or without cirrhosis. The results revealed the significant diagnostic values of *PF4* and *PPBP* in cirrhotic HCC, and *CCL19*, *CCL25* in non-cirrhotic HCC, while no significant diagnostic values of *CNR1* in HCC with or without cirrhosis were found (Fig. 4). Finally, Clinical characteristics analysis showed that high expression of *CNR1* was greatly correlated with less vascular invasion and fewer HBV infection in non-cirrhotic HCC (Table 2), while high *CCL19* expression was positively associated with HBV infection in non-cirrhotic (Table 3). No significant clinical characteristics of *PF4*, *PPBP* were found in cirrhotic HCC, neither *CCL25* in non-cirrhotic HCC.

**Table 2:** Correlations the clinical characteristics among CNR1 between HCC with or without cirrhosis in TCGA

Variables	N	Non-cirrhotic HCC		N	Cirrhotic HCC	
		CNR1			CNR1	
		Low/high expression case (n)	p value		Low/high expression case (n)	p value
Gender						
Male	82	44/38	0.282	61	31/30	0.635
Female	50	22/28		18	8/10	
Age at diagnosis						
≥ 60	80	38/42	0.476	36	17/19	0.650
< 60	52	28/24		42	22/20	
NA	0	0		1	0/1	
T stage						
T1/T2	97	48/49	1.000	70	35/35	0.712
T3/T4	33	17/16		8	3/5	
TX	1	1/0		0	0/0	
NA	1	0/1		1	1/0	
N stage						
N0	91	49/42	0.259	57	29/28	1.000
N1	1	0/1		1	0/1	
NX	40	17/23		20	10/10	
NA	0	0/0		1	0/1	
M stage						
M0	90	46/44	0.529	62	31/31	0.830
M1	4	3/1		0	0/0	
MX	38	17/21		17	8/9	
Pathologic stage						
I/II	91	44/47	0.755	67	35/32	0.478
III/IV	33	17/16		9	3/6	
NA	8	5/3		3	1/2	
Vascular invasion						
None	87	38/49	0.127	52	26/26	0.900
Micro	33	18/15		22	10/12	
Macro	6	5/1		2	1/1	
NA	6	5/1		3	2/1	
HBV infection						
Yes	38	25/13	*0.036*	36	20/16	0.358
No	88	40/48		40	18/22	
NA	6	1/5		3	1/2	
HCV infection						
Yes	14	6/8	0.488	20	13/7	0.118
No	112	59/53		56	25/31	
NA	6	1/5		3	1/2	

*NA* not applicable

The significant p value is marked with italic type

**Table 3:** Correlations the clinical characteristics among *CCL19* and non-cirrhotic HCC in TCGA

Variables	N	Non-cirrhotic HCC	
		*CCL19*	
		Low/high expression case (n)	p value
Gender			
Male	82	64/18	0.585
Female	50	41/9	
Age at diagnosis			
≥ 60	80	68/12	0.054
< 60	52	37/15	
T stage			
T1/T2	97	76/21	0.846
T3/T4	33	27/6	
TX	1	1/0	
NA	1	1/0	
N stage			
N0	91	73/18	0.853
N1	1	1/0	
NX	40	31/9	
M stage			
M0	90	74/16	0.178
M1	4	2/2	
MX	38	29/9	
Pathologic stage			
I/II	91	72/19	0.741
III/IV	33	27/6	
NA	8	6/2	
Vascular invasion			
None	87	69/18	1.000
Micro	33	26/7	
Macro	6	5/1	
NA	6	5/1	
HBV infection			
Yes	38	27/11	0.176
No	88	72/16	
NA	6	6/0	
HCV infection			
Yes	14	7/7	*0.016*
No	112	92/20	
NA	6	6/0	

*NA* not applicable

The significant p value is marked with italic type

### Construct differentially miRNA-mRNA networks regulate in HCC with or without cirrhosis

**Fig. 5:**
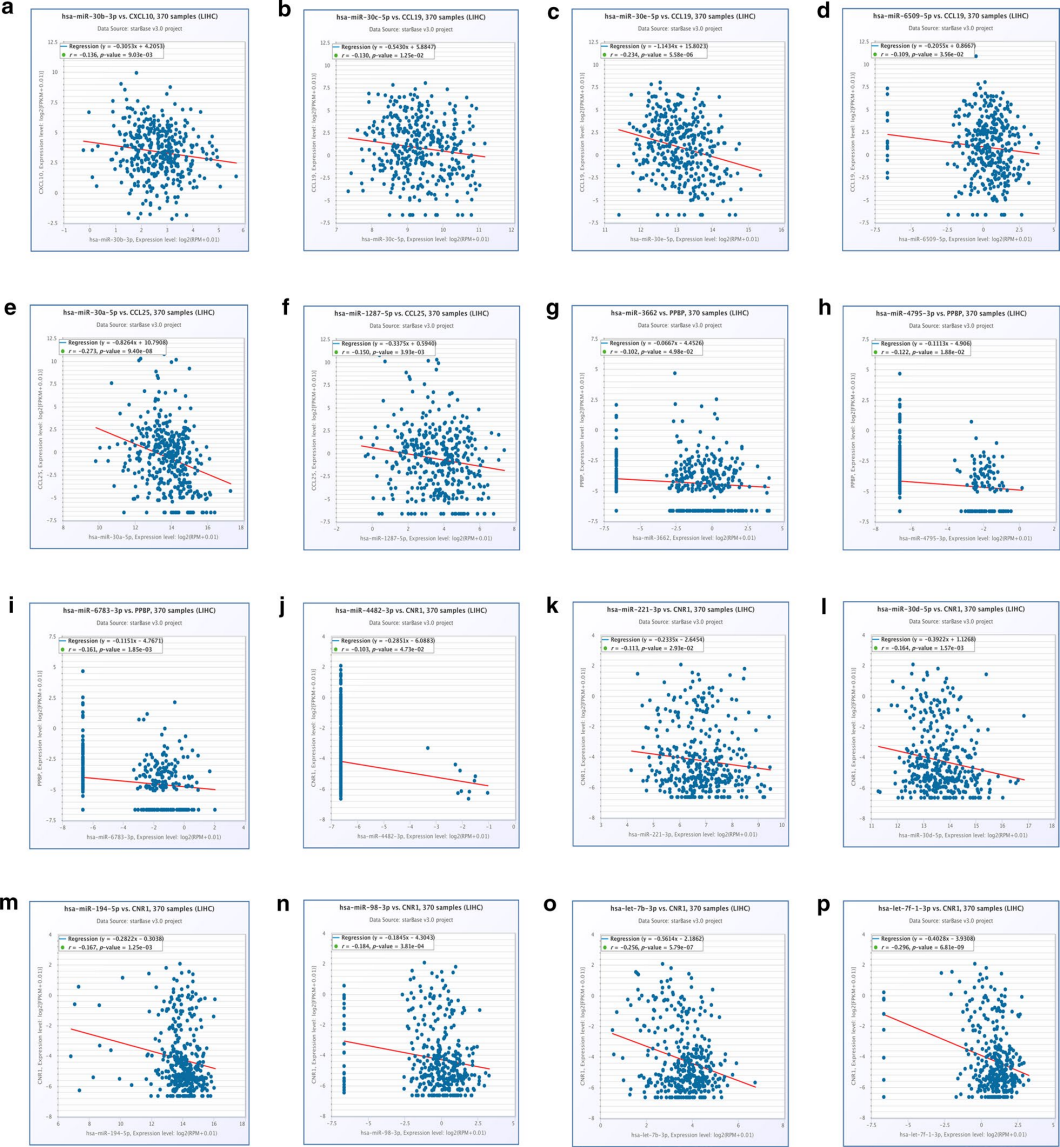
Correlation analysis between 517 candidate target miRNAs from miRDB database and 7 key DEGs by using starBase database (only 16 significant correlation analysis were showed). **a** hsa-miR-30a-5p vs. *CCL25*; **b** hsa-miR-1287-5p vs. *CCL25*; **c** hsa-miR-3662 vs. *PPBP*; **d** hsa-miR-4795-3p vs. *PPBP*; **e** hsa-miR-6783-3p vs. *PPBP*; **f** hsa-miR-4482-3p vs. *CNR1*; **g** hsa-miR-221-3p vs. *CNR1*; **h** hsa-miR-30d-5p vs. *CNR1*; **i** hsa-miR-194-5p vs. *CNR1*; **g** hsa-miR-98-3p vs. *CNR1*; **k** hsa-let-7b-3p vs. *CNR1*; **l** hsa-let-7f-1-3p vs. *CNR1*; **m** hsa-miR-30c-5p vs. *CCL19*; **n** hsa-miR-30e-5p vs. *CCL19*; **o** hsa-miR-6509-5p vs. *CCL19*

**Fig. 6:**
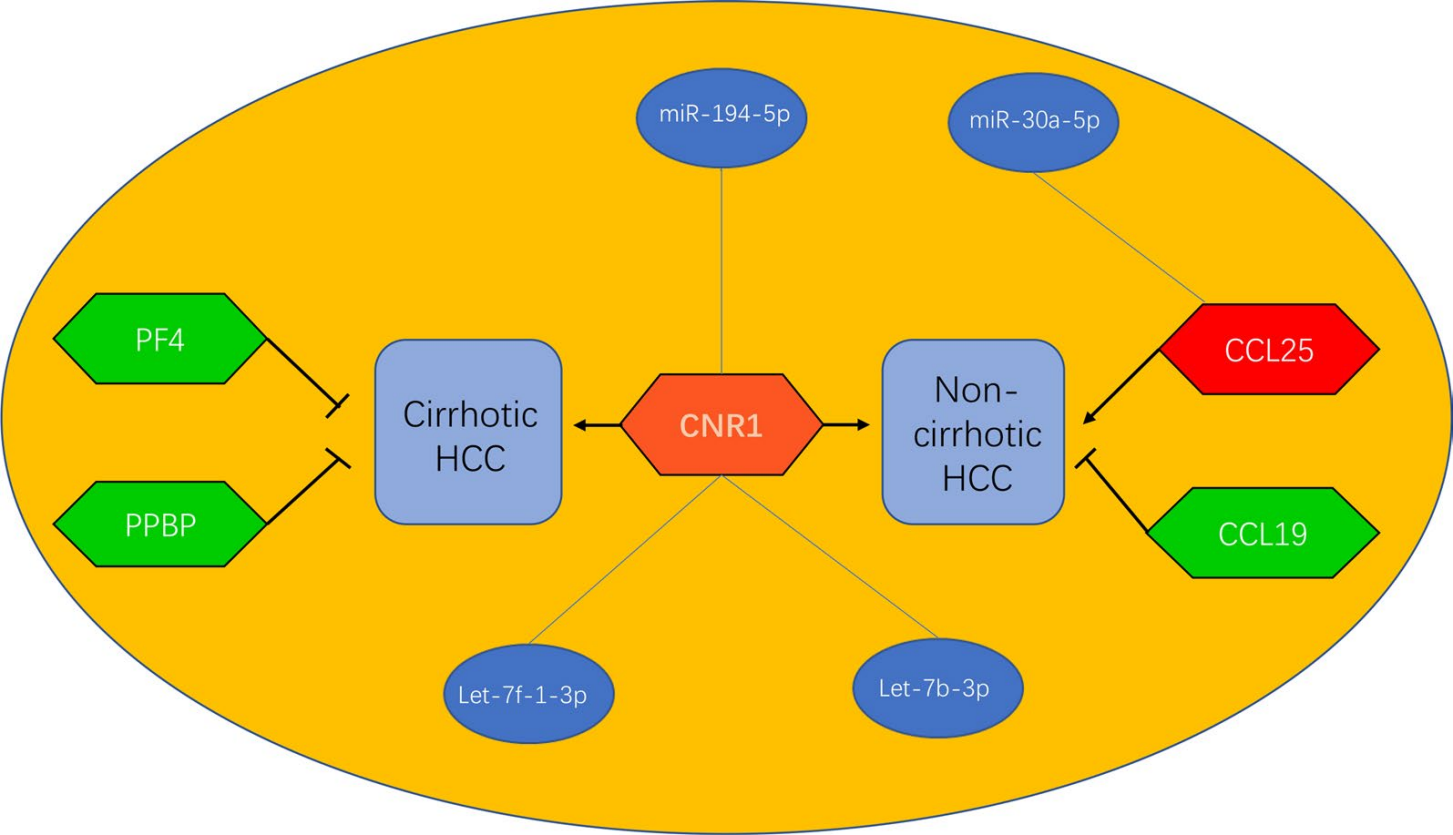
miRNA-mRNA regulate networks constructed in non-cirrhotic HCC and cirrhotic HCC

To find the regulatory mechanisms about non-cirrhotic HCC and cirrhotic HCC, the miRDB database was employed to predict the microRNAs (miRNAs) which could regulate 5 target genes. Besides, we intended to further evaluate the correlation between predicted miRNAs and corresponding target genes using the starBase database. As we all know, target genes were conversely regulated by miRNAs, and the higher correlation coefficient (r), the greater mutual regulation effect. Thus, we chose those predicted miRNAs that negatively correlated with target genes and the |r| > 0.1, p-value < 0.05 for the next research. The analytic results showed in Fig. 5 indicated that *CCL19* could be potentially modulated by miR-30c-5p, miR-30e-5p and miR-6509-5p. miR-30a-5p and miR-1287-5p could potentially target *CCL25*. In addition, miR-3662, miR-4795-3p and miR-6783-3p could potentially target *PPBP*. No predicted miRNAs of *PF4* met the conditions. As for *CNR1*, 7 miRNAs including miR-4482-3p, miR-221-3p, miR-30d-5p, miR-194-5p, miR-98-3p, let-7b-3p and let-7f-1-3p were selected as potential negative regulators. After that, we analyzed those miRNAs expression between corresponding normal liver tissues and HCC tissues with or without cirrhosis in GSE10694 database from GEO, which was included 40 cirrhotic HCC patients and 38 non-cirrhotic HCC patients [31]. Based on this analysis, we found that miR-194-5p was downregulated in HCC tissues with or without cirrhosis than corresponding normal liver tissues. The expression level of miR-30c-5p was downregulated in non-cirrhotic HCC patients compared with corresponding non-cirrhotic normal liver tissues, while miR-30e-5p and miR-30a-5p were also downregulated in non-cirrhotic HCC tissues compared with corresponding non-cirrhotic normal liver tissues, not as expected (not list Table 4). Besides, let-7b-3p and let-7f-1-3p expression were higher in cirrhotic normal liver tissues than in cirrhotic HCC tissues (Table 4). Finally, combined with the expression levels of the key miRNAs between HCC with or without cirrhosis and normal tissues, the key miRNA-mRNA regulatory networks in non-cirrhotic HCC and cirrhotic HCC were constructed (Fig. 6).

**Table 4:** Key miRNAs significantly differentially expressed in cirrhotic HCC or cirrhotic HCC compared with corresponding normal liver tissues in GSE10694 from GEO database

GSE10694	adj.p.Val	logFC	miRNA_ID	Target gene
Non-cirrhotic HCC				
Downregulated	3.43E−01	0.2545	hsa-miR-194-5p	*CNR1*
Downregulated	9.32E−02	0.29473	hsa-miR-30c-3p	*CCL19*
Cirrhotic HCC				
Downregulated	5.72E−01	0.20674	hsa-miR-194-5p	*CNR1*
Downregulated	6.73E−01	0.17521	hsa-let-7f-1-3p	*CNR1*
Downregulated	6.90E−01	0.15632	hsa-let-7b-3p	*CNR1*

*NA* not applicable

**Fig. 7:**
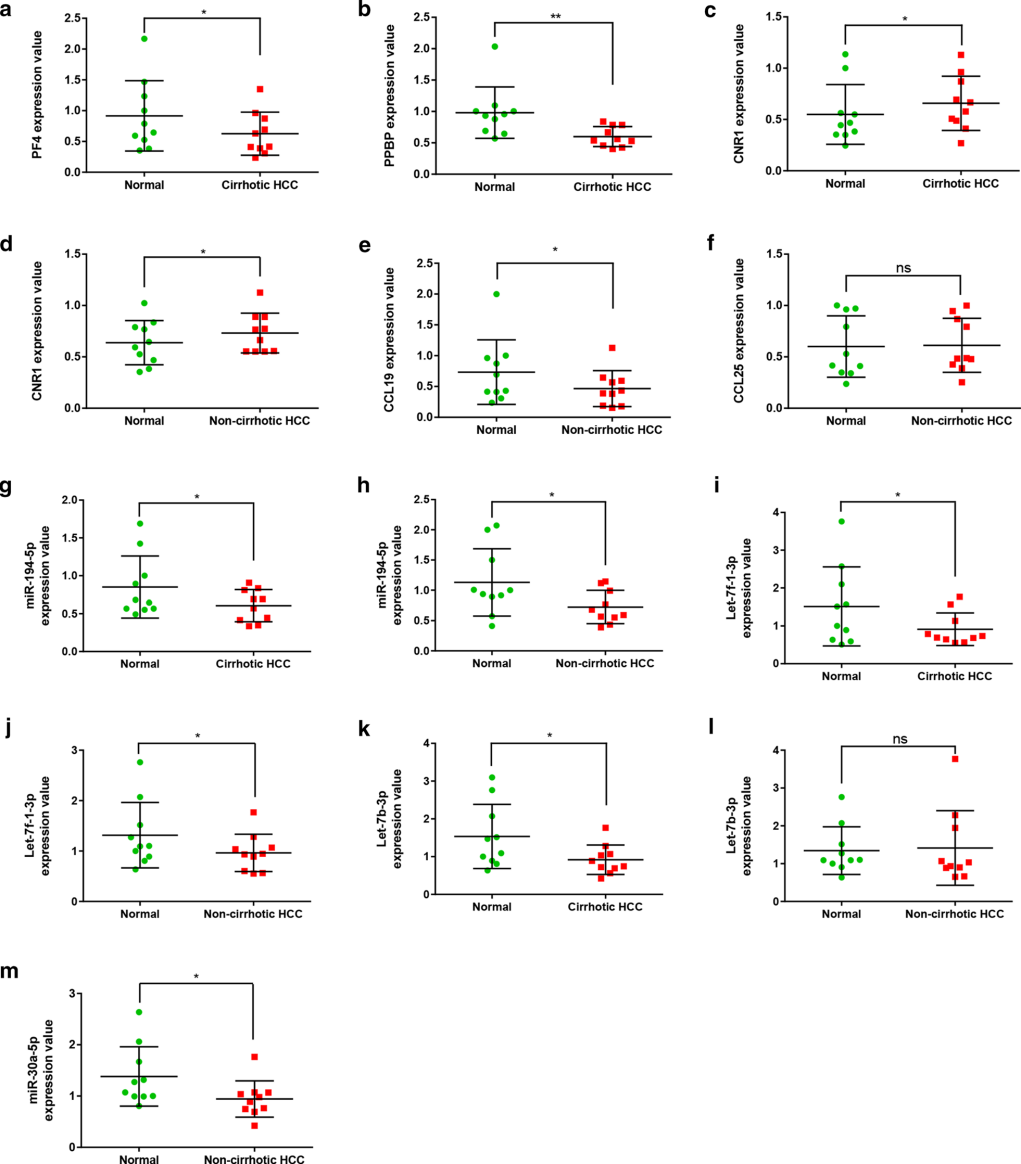
The expression levels of potential miRNAs and target genes in cirrhotic HCC or non-cirrhotic HCC tissues compared to matched normal tissues. **a** *PF4* expression between cirrhotic HCC and normal tissues; **b** *PPBP* expression between cirrhotic HCC and normal tissues; **c** *CNR1* expression between cirrhotic HCC and normal tissues; **d** *CNR1* expression between non-cirrhotic HCC and normal tissues; **e** *CCL19* expression between non-cirrhotic HCC and normal tissues; **f** *CCL25* expression between non-cirrhotic HCC and normal tissues; **g** miR-194-5p expression between cirrhotic HCC and normal tissues; **h** miR-194-5p expression between non-cirrhotic HCC and normal tissues; **i** let-7f-1-3p expression between cirrhotic HCC and normal tissues; **g** let-7f-1-3p expression between non-cirrhotic HCC and normal tissues; **k** let-7b-3p expression between cirrhotic HCC and normal tissues; **l** let-7b-3p expression between non-cirrhotic HCC and normal tissues; **m** miR-30a-5p expression between non-cirrhotic HCC and normal tissues

To validate the values of these key miRNAs and target genes, we further detected the expression levels in 20 pairs of HCC clinical samples (10 cirrhotic HCC and 10 non-cirrhotic HCC) and compared with corresponding normal liver tissues. As the results shown in Fig. 7, qRT-PCR indicated that *PF4* and *PPBP* were downregulated in cirrhotic HCC tissues compared with normal liver tissues. *CNR1* was upregulated in both cirrhotic HCC and non-cirrhotic HCC tissues. Besides, *CCL19* was downregulated in non-cirrhotic HCC, while no significant value of *CCL25* was found between non-cirrhotic HCC and corresponding normal liver samples. In addition, miR-194-5p and let-7f-1-3p were downregulated in HCC with or without cirrhosis than in paracancerous tissues. What’s more, let-7b-3p was downregulated in cirrhotic HCC compared to matched normal liver tissues, while not in non-cirrhotic HCC. Additionally, compared with normal liver tissues, the expression levels of miR-30a-5p was significantly decreased in non-cirrhotic HCC. All these findings suggested that miR-194-5p/*CNR1* and let-7f-1-3p/*CNR1* axes may play important roles in both non-cirrhotic HCC and cirrhotic HCC. Let-7b-3p/*CNR1* axis may exert biological effect in cirrhotic HCC. Furthermore, *CCL19* and miR-30a-5p were also worth to research in non-cirrhotic HCC, as well as *PPBP* and *PF4* in cirrhotic HCC.

## Discussion

As the most common malignancy in the liver, hepatocellular carcinoma includes two kinds of types, non-cirrhotic HCC and cirrhotic HCC. HCC patients with or without cirrhosis present different clinical characteristics, and regulated by various miRNA-mRNA networks. However, few researches directly investigated the underlying molecular mechanisms between cirrhotic HCC and non-cirrhotic HCC. Therefore, it’s meaningful to explore the differences between two kinds of types in HCC.

In this study, we discovered DEGs between cirrhotic HCC and non-cirrhotic HCC by performing a differential expression analysis based on the data from TCGA. Next, by comparing 15 hub genes expression levels in the cirrhotic HCC or non-cirrhotic HCC tissues with corresponding normal tissues expression levels in TCGA, we found 5 key DEGs between two kinds of types in HCC. Furthermore, after validating the expression of predicted miRNAs between HCC (cirrhosis or non-cirrhosis) and corresponding normal liver tissues (cirrhosis or non-cirrhosis) in GEO database, we selected key predicted miRNAs for 5 key genes and first constructed the miRNA-mRNA regulatory networks in HCC with or without cirrhosis. In the next step, we further analyzed the expression levels of these key miRNAs and genes in clinical samples.

Our study first reported that high expression of *CNR1* was associated with worth OS in cirrhotic HCC but not in non-cirrhotic HCC, and correlated with less vascular invasion and fewer HBV infection in non-cirrhotic HCC. Besides, *CNR1* expression was upregulated in HCC with or without cirrhosis than that in compared normal tissues both in TCGA and qRT-PCR results. What’s more, Liu et al. [32] demonstrated that loss of cannabinoid receptor 1 (*CNR1*) expression could reduce liver-specific gene expression, thus leading to smaller livers. Additionally, our analytic results showed that *CNR1* was an independent risk indicator for the prognosis of cirrhotic HCC patients but not of non-cirrhotic HCC patients, indicating that two kinds of HCC types may involve separate risk factors. Meanwhile, *CNR1* might be an important tumor promoter in HCC. It’s unexpected that *CNR1* expression was higher in non-cirrhotic HCC tissues than corresponding normal tissues in TCGA, whereas associated with less vascular invasion and fewer HBV infection in non-cirrhotic HCC. Thus, additional studies of *CNR1* are necessary to investigate the expression and function in HCC including cirrhotic HCC and non-cirrhotic HCC in the future.

What’s more, ROC analysis revealed that *CCL19*, *CCL25* may play important roles in non-cirrhotic HCC diagnosis and *PF4*, *PPBP* have important diagnostic values in cirrhotic HCC, but no diagnostic values of *CNR1* in cirrhotic HCC and non-cirrhotic HCC were found. Clinical characteristics analysis revealed that higher expression level of *CCL19* was associated with higher HCV infection. QRT-PCR results found important values of *CCL19* in non-cirrhotic HCC, *PF4* and *PPBP* in cirrhotic HCC, while without CCL25. All of the results indicated that these genes play important roles in HCC diagnosis, and worth to research in HCC with or without cirrhosis. It’s also surprising to find that let-7f-1-3p and let-7b-3p which were predicted to target *CNR1* have no significant differentially expressed values between non-cirrhotic HCC tissues and corresponding normal liver tissues in GEO database. Because of limited clinical data, more studies are needed to investigate this.

After reviewing previous articles, we found that lots of researches in HCC have already been launched to investigate the roles of the mRNAs and miRNAs involved in the constructed networks. For example, *CCL25* could promote invasion and migration of HCC [33]. Qin et al. [34] indicated that miR-30e-5p could inhibit HBV replication. Besides, let-7b-3p and miR-30c-5p may be useful biomarkers of early hepatitis B virus-related HCC and HCV-related HCC patients, respectively [35, 36]. Whereas, *CCL19* inhibited the proliferation and migration ability of HCC cells [37]. miR-194-5p accelerated the progression of HBV-related liver disease [38]. Although PF4 does not seem to be a prognostic or diagnostic biomarker in HCC for cirrhotic patients [39], our research found that PF4 may play an important role in cirrhotic-HCC. However, the functions of *PPBP*, *CCL25*, miR-30a-5p and let-7f-1-3p in HCC are still not clearly. Furthermore, researches about the mRNAs and miRNAs involved in constructed networks aiming at specific types of HCC (cirrhotic HCC or non-cirrhotic HCC) are still extremely limited.

It’s obvious that differential miRNA-mRNA regulatory networks are greatly participated in HCC progression including cirrhotic HCC and non-cirrhotic HCC. Those key genes and miRNAs in our study are closely related with prognostic values, diagnostic values and some specific clinical characteristics in HCC patients, which can make us better understand the mechanisms between non-cirrhotic HCC and cirrhotic HCC and provide effective and promising approaches in treating HCC. However, there are still some limitations in this study, such as (1) only top 15 hub genes was validated for the further research; (2) lack of research on detailed molecular mechanisms that the key mRNAs and key miRNAs regulate in HCC with or without cirrhosis; (3) some function studies about mRNAs and miRNAs in the constructed networks were not as excepted, and lots of mRNAs and miRNAs were lack of research. Even so, our finding remains useful, as the differential miRNA-mRNA regulatory networks in HCC patients with or without cirrhosis may provide more helpful biomarkers, improve prognosis and therapy of HCC patients in further.

## Conclusion

In conclusion, we first successfully constructed the miRNA-mRNA networks between cirrhotic HCC and non-cirrhotic HCC by using bioinformatic analysis and preliminary experimental validation. The present study suggested that *CNR1*, negatively regulated by miR-194-5p and let-7f-1-3p, might be a promising factor of cirrhotic and non-cirrhotic HCC patients. Let-7b-3p may regulate *CNR1* participant in cirrhotic HCC progression. miR-30a-5p-*CCL25* axis may play an important role in promoting non-cirrhotic HCC progression. In addition, *PF4* and *PPBP* may function as tumor suppressors in cirrhotic HCC, meanwhile, *CCL19* may act as a protective factor in non-cirrhotic HCC, while miRNAs regulate mechanisms still unclear, further experimental trials are still need to be launched in the future.

## Additional files

**Additional file 1: Table S1.** Primers of key mRNAs.

**Additional file 2: Figure S1.** PPI network of DEGs between cirrhotic HCC and non-cirrhotic HCC by using STRING database.

## Data Availability

Not applicable.
