# Supplementary material for: In silico analysis excavates potential biomarkers by constructing miRNA-mRNA networks between non-cirrhotic HCC and cirrhotic HCC
Source: Cancer Cell Int. 2019 Jul 18;19:186. doi: 10.1186/s12935-019-0901-3 (PMC6637578; doi:10.1186/s12935-019-0901-3)
Supplement: Supplementary file 1 — Additional file 1: Table S1. Primers of key mRNAs. [file 12935_2019_901_MOESM1_ESM.docx]

**Table S1**

| **mRNA** | **primers** |
| --- | --- |
| ***CNR1*** | Forward Primer: ATGTGGACCATAGCCATTGTG  Reverse Primer: CCGATCCAGAACATCAGGTAGG |
| ***CCL19*** | Forward Primer: CTGCTGGTTCTCTGGACTTCC  Reverse Primer: AGGGATGGGTTTCTGGGTCA |
| ***CCL25*** | Forward Primer: GGCCCTCATGCTGTAAAGAAG  Reverse Primer: TGCTGATGGGATTGCTAAACTT |
| ***PF4*** | Forward Primer: AAGCCCCGCTGTACAAGAAA  Reverse Primer: TATATAGCAAATGCACACACGTAG |
| ***PPBP***  ***GAPDH*** | Forward Primer: GAACTCCGCTGCATGTGTATAA  Reverse Primer: GCAATGGGTTCCTTTCCCGAT  Forward Primer: CTGGGCTACACTGAGCACC  Reverse Primer: AAGTGGTCGTTGAGGGCAATG |
