# Supplementary figures and images for: In silico analysis excavates potential biomarkers by constructing miRNA-mRNA networks between non-cirrhotic HCC and cirrhotic HCC
Source: Cancer Cell Int. 2019 Jul 18;19:186. doi: 10.1186/s12935-019-0901-3 (PMC6637578; doi:10.1186/s12935-019-0901-3)

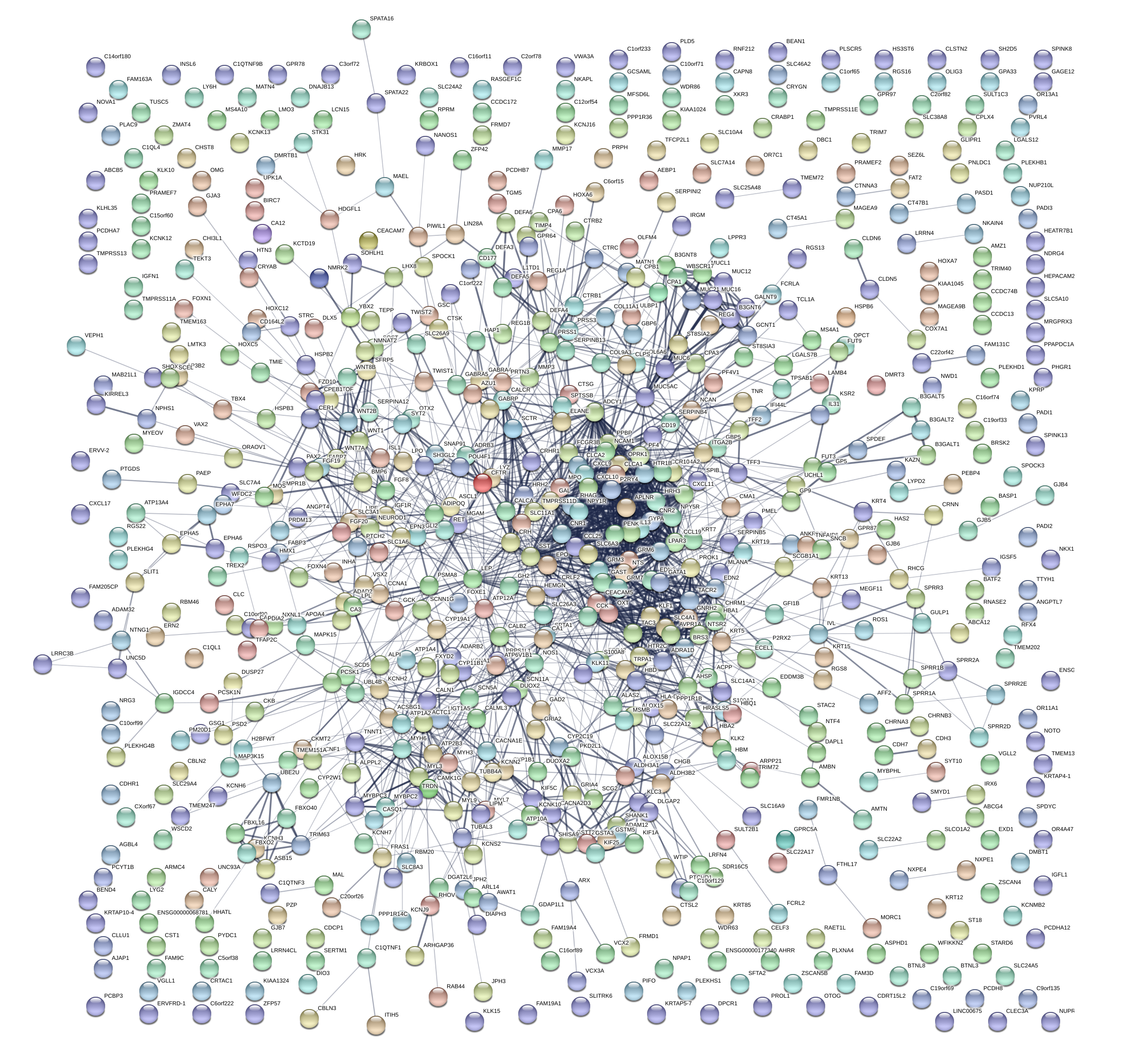

Supplement: Supplementary file 2 — Additional file 2: Figure S1. PPI network of DEGs between cirrhotic HCC and non-cirrhotic HCC by using STRING database. [file 12935_2019_901_MOESM2_ESM.tif]
